# Prevalence and Risk Factors for Low Bone Mineral Density in Adults With Cystic Fibrosis

**DOI:** 10.1002/jbm4.10666

**Published:** 2022-09-16

**Authors:** Rebecca L Boyle, Kevin J Psoter, Christian A Merlo, Aniket R Sidhaye, Noah Lechtzin, Shivani Patel, Kristina Montemayor, Alexandra Horne, Natalie E West

**Affiliations:** ^1^ Division of Pulmonary and Critical Care Medicine Johns Hopkins University Baltimore MD USA; ^2^ Division of General Pediatrics Johns Hopkins University Baltimore MD USA; ^3^ Division of Endocrinology Johns Hopkins University Baltimore MD USA

**Keywords:** BONE MINERAL DENSITY, CYSTIC FIBROSIS, CYSTIC FIBROSIS–RELATED BONE DISEASE, DXA, SCREENING

## Abstract

Single‐center studies have suggested that up to 70% of adults with cystic fibrosis (CF) have lower than expected bone mineral density (BMD), substantially higher than the 25% prevalence reported from national registries. We determined the prevalence of low BMD in CF adults at our center and assessed risk factors for low BMD. This retrospective cohort study was conducted in all CF patients ≥18 years of age who had a dual‐energy X‐ray absorptiometry (DXA) scan performed at the Johns Hopkins Adult Cystic Fibrosis center between 2010 and 2018. Prevalence and incidence of low BMD during the study period were determined. Poisson regression based on generalized estimating equations and robust standard errors were used to evaluate selected risk factors and risk of disease progression. A total of 234 individuals underwent an initial DXA scan. At this scan, prevalence of low BMD was 52.6% (95% confidence interval [CI] 46.0–59.1). A total of 43.6% were at risk for CF‐related low BMD (AR‐CFLBMD) (95% CI 37.1–50.2) and 9.0% had CF‐related low BMD (CFRLBMD) (95% CI 5.6–13.4). Of the 25 with normal BMD at initial scan and a subsequent follow‐up scan, 8 (32.0%) progressed to AR‐CFLBMD. Of the 53 with AR‐CFLBMD on initial scan and a subsequent scan, 6 (11.3%) progressed to CFLBMD, 9 (17.0%) returned to normal BMD, and 38 (71.7%) remained AR‐CFLBMD. Older age (relative risk [RR] = 1.01; 95% CI 1.00–1.01) and male sex (RR = 1.32; 95% CI 1.04–1.66) were associated with increased risk of low BMD, while higher forced expiratory volume over 1 second (FEV_1_%) predicted (RR = 0.99; 95% CI 0.99–1.00) and body mass index (BMI; RR = 0.97; 95% CI 0.94–1.00) were associated with lower risk for low BMD. The fact that more than half of all individuals were found to have lower than expected BMD suggests that the actual prevalence may be higher than currently reported in national registries. This supports the importance of universal bone health screening of all CF adults. © 2022 The Authors. *JBMR Plus* published by Wiley Periodicals LLC on behalf of American Society for Bone and Mineral Research.

## Introduction

As the number of adults with cystic fibrosis (CF) has grown,^(^ ^1^ ^)^ so has the recognition of the increased risk of low bone mineral density (BMD) in CF adults.^(^ ^2^ ^)^ American, French, and UK registries report low BMD prevalence among adults with CF as 25.0%,^(^ ^1^ ^)^ 25.2%,^(^ ^3^ ^)^ and 24.2%,^(4^ ^)^ respectively. This likely represents a significant underestimation attributable to inadequate screening, as single‐center studies have suggested that up to 70% of adults with CF may have low BMD.^(^ ^2^, ^5^, ^6^, ^7^ ^)^ Although current guidelines published by the Cystic Fibrosis Foundation (CFF)^(^ ^8^ ^)^ and European Cystic Fibrosis Society (ECFS)^(^ ^9^ ^)^ recommend that dual‐energy X‐ray absorptiometry (DXA) assessment of BMD be obtained on all adults with CF, and the International Society for Clinical Densitometry (ISCD) recommends that all adults with diseases or conditions associated with low bone mass undergo BMD testing, a significant percentage of CF adults do not receive screening DXA scans.^(^ ^1^ ^)^ Despite the fact that a diagnosis of low BMD does not directly relate to individual fracture risk, it is highly predictive for fragility fractures in the future.^(^ ^10^ ^)^ Low BMD is of particular concern in CF because increased risk of rib^(^ ^11^ ^)^ and spine^(^ ^12^ ^)^ fractures can lead to reduced airway clearance and exercise ability, as well as chronic pain. In addition, low BMD can lead to significant challenges in people with CF requiring lung transplantation^(^ ^13^ ^)^ as transplantation medications, such as glucocorticoids, frequently lead to progressive worsening of BMD status.^(^ ^14^, ^15^ ^)^ Given the morbidity associated with low BMD, additional data on the true prevalence of low BMD in CF adults are needed to better manage the manifestations of low BMD.

Previous studies have identified risk factors associated with low BMD in people with CF, including low lung function, poor nutritional status, decreased absorption of vitamin D, altered sex hormone production, chronic lung infection, physical inactivity, glucocorticoid therapy, delayed puberty or hypogonadism, and CF‐related diabetes (CFRD).^(^ ^8^, ^9^, ^16^, ^17^, ^18^ ^)^ Additional data identifying which of these factors most strongly correlates with low BMD would help to more accurately identify higher‐risk CF populations that could receive particular attention to their BMD status and early therapeutic intervention. With the primary aim of providing additional data on prevalence of low BMD in CF adults and the secondary aim of evaluating risk factors for low BMD, we performed a retrospective review of all adult CF patients seen at Johns Hopkins who had DXA scans during the study period. We hypothesize that the prevalence of individuals with low BMD is greater than the 25% reported in registries. We also expect that the risk factors for low BMD correspond to those identified in previous studies, specifically, low lung function and poor nutritional status.

## Materials and Methods

### Study design and patient population

This was a retrospective cohort study conducted in all CF patients ≥18 years of age seen at the Johns Hopkins Adult CF Center who had a DXA scan performed between 2010 and 2018. Data were collected via chart review of the electronic medical record and local CFF registry. Patients with a history of lung transplantation were censored at the year of transplant but could contribute observations up until the year in which the transplant took place. The study protocol was reviewed and approved by the Johns Hopkins School of Medicine Institutional Review Board.

### Measurements

All DXA scans within the study period were reviewed; those before the study period (before 2010) were not included. The type of DXA scanner model was collected. Demographic and clinical data were collected at the time that the initial DXA scan was performed during the study period and at each subsequent scan. These data included age, sex, race (white or nonwhite), genotype (homozygous F508del, heterozygous F508del, or other), pancreatic insufficiency status based on pancreatic enzyme oral supplementation, diagnosis of CFRD, and diagnosis of CF‐related liver disease (CFLD). Highest lung function (forced expiratory volume over 1 second [FEV_1_]% predicted) and body mass index (BMI) in the year before the DXA exam were recorded. 25‐hydroxyvitamin D (25OHD), creatinine, and calcium serum levels in the year before the DXA exam were also recorded (the most recent value recorded if multiple values were obtained in that year).

Cystic fibrosis transmembrane conductance regular (CFTR) modulator use was also determined at the time of first DXA exam by treatment with ivacaftor, lumacaftor/ivacaftor, or tezacaftor/ivacaftor (elexacaftor/tezacaftor/ivacaftor was not yet approved during the study period).

BMD was assessed for both total hip and lumbar spine, and *T*/*Z*‐scores were obtained from the reports of existing DXA scans. Results were categorized as normal BMD (*T*/*Z*‐score greater than −1), at risk for progression to CF‐related low BMD (AR‐CFLBMD) (*T*/*Z*‐score between −1.0 and − 2.0), or CF‐related low BMD (CFLBMD) (*T*/*Z*‐score ≤ −2.0) according to current CFF and ECFS guidelines regarding low BMD treatment and follow‐up.^(^ ^8^ ^)^ The ECFS labels *T*/*Z*‐scores ≤ −2.0 in CF adults as “CFLBMD,”^(^ ^19^ ^)^ but there is no standard nomenclature describing individuals with −1.0 > *T*/*Z*‐score > −2.0. Therefore, we have termed this group “AR‐CFLBMD” to emphasize their risk of progression to CFLBMD. The primary outcome for this study was lower than expected BMD defined as AR‐CFLBMD or CFLBMD, with secondary outcome risk factors for lower than expected BMD in CF adults.

Current WHO and ISCD statements define osteoporosis in postmenopausal women and men over the age of 50 years as a DXA of the lumbar spine, total hip, or femoral neck with a *T*‐score ≤ −2.5.^(^ ^20^ ^)^ Although the WHO defines osteopenia as −1.0 ≥ *T*‐score ≥ −2.5,^(^ ^20^ ^)^ the ISCD states that “low bone mass” or “low bone density” are preferred over “osteopenia.”^(^ ^21^ ^)^ In premenopausal females and males under the age of 50 years, a *Z*‐score ≤ −2.0 is considered lower than the expected range and scores ≥ −2.0 are considered within the expected range.^(^ ^21^ ^)^ A diagnosis of osteoporosis is uncommon in premenopausal women and in men under the age of 50 years and often requires the presence of a fragility fracture.^(^ ^21^, ^22^ ^)^ In this study, we chose to follow CFF and ECFS guidelines for bone health care in CF adults as described above.

### Statistical analysis

Demographic and clinical characteristics of the study population were summarized and compared between those with and without low BMD at initial DXA scan using Student's *t* and chi‐square or Fisher exact tests for continuous and categorical variables, respectively.

Prevalence and binomial exact 95% confidence intervals (CI) of lower than expected BMD, AR‐CFLBMD, and CFRBMD was determined based on initial DXA scan results. Incidence of AR‐CFLBMD and CFLBMD was based upon those individuals with a normal initial DXA scan who had a subsequent follow‐up scan.

Because of the non‐rare nature of the outcome and to utilize all DXA scan results during follow‐up, unadjusted and adjusted generalized estimating equations (GEE)–based Poisson regression with robust standard errors was used to evaluate selected risk factors and risk of low BMD. Risk factors assessed were chosen a priori and included age at DXA scan, sex, race, F508del genotype, pancreatic insufficiency, highest FEV_1_% predicted, and BMI in the year before DXA scan and CFTR modulator use. Results of regression models are reported as relative risks (RR) with corresponding 95% CI. A *p* value <0.05 was considered statistically significant. All analyses were performed using STATA Version 16.1 (College Station, TX, USA).

## Results

### Participant characteristics

A total of 234 (65.0%) of 360 pre‐transplant adult CF patients underwent at least one DXA scan between 2010 and 2018. Table 1 presents the demographic and clinical characteristics of the study population overall and by BMD status (normal or low) at time of initial DXA scan. Mean age at time of initial DXA scan was 32.9 (SD = 11.88) years. The majority of individuals receiving a DXA scan were female (54.1%) and white (93.0%). A total of 43.4% were homozygous for F508del, 82.8% were pancreatic insufficient, 30.3% had CFRD, and 12.4% had CFLD. The mean FEV_1_% predicted was 69.3 ± 24.1%. Mean BMI was 24.2 ± 5.0 kg/m^2^ and mean 25OHD 31.3 ± 13.6 ng/mL. Mean serum calcium level was 9.05 ± 0.82 mg/dL and mean serum creatinine was 0.8 ± 0.2 mg/dL. A total of 54.7% were on a CFTR modulator at any point in the study period, with 7.3% being on ivacaftor, 6.4% being on lumacaftor/ivacaftor, and 0.4% being on tezacaftor/ivacaftor at the time of the initial DXA. A total of 9.8% of individuals were on bisphosphonate therapy before their first DXA scan and 7.6% of individuals with repeat DXA scans started bisphosphonate therapy during the course of the study. Compared with individuals with normal BMD, a greater proportion of individuals with lower than expected BMD were male (54.5 versus 36.4%). The average FEV_1_% predicted (62.8 versus 76.6) and BMI (23.5 versus 24.9) of those with low BMD in the year before initial scan were lower than those with normal BMD. Seven participants had fragility fractures before their first DXA scan; 3 fractures were in individuals with normal BMD and 4 were in individuals with AR‐CFLBMD (none were in individuals with CFLBMD).

**Table 1:** Baseline Study Population Characteristics

	Overall (*n* = 234)	Normal BMD (*n* = 111)	Lower than expected BMD (*n* = 123)	*p* Value
Age, mean (SD)	32.9 (11.88)	31.5 (10.17)	34.2 (13.10)	0.089
Male, *n* (%)	107 (45.9)	40 (36.4)	67 (54.5)	0.006
Race, non‐White, *n* (%)	16 (7.0)	5 (4.6)	11 (9.1)	0.180
Genotype, *n* (%)				0.945
Homozygous F508del	99 (42.3)	48 (43.2)	51 (41.5)	
Heterozygous F508del	104 (44.4)	49 (44.1)	55 (44.7)	
Other	31 (13.3)	14 (12.6)	17 (13.8)	
Pancreatic insufficiency, *n* (%)	192 (82.8)	86 (78.9)	106 (86.2)	0.143
CF‐related diabetes mellitus, *n* (%)	71 (30.3)	34 (30.6)	37 (30.1)	0.92
CF‐related liver disease, *n* (%)	29 (12.4)	12 (10.8)	17 (13.8)	0.81
FEV_1_% predicted, mean (SD)	69.3 (24.09)	76.6 (22.43)	62.8 (23.74)	<0.001
FEV_1_% predicted, *n* (%)				<0.001
>50	167 (76.3)	90 (87.4)	77 (66.4)	
30–50	42 (19.2)	12 (11.7)	30 (25.9)	
<30	10 (4.6)	1 (1.0)	9 (7.8)	
BMI, mean (SD)	24.2 (4.95)	24.9 (5.67)	23.5 (4.11)	0.027
Fragility fracture, *n* (%)	7 (3.0)	3 (2.7)	4 (3.3)	
CFTR modulator use, *n* (%)	35 (15.00	18 (16.2)	17 (13.8)	0.608
Bisphosphonate use, *n* (%)	23 (9.8)	2 (8.1)	21 (17.1)	<0.001
25OHD, mean (SD)	31.3 (13.57)	29.4 (12.29)	32.8 (14.39)	0.157
Calcium, mean (SD)	9.05 (0.82)	9.2 (0.4)	9 (1.0)	0.1
Creatinine, mean (SD)	0.9 (0.65)	0.8 (0.2)	1 (0.8)	0.09

BMD = bone mineral density; SD = standard deviation; CF = cystic fibrosis; FEV_1_ = forced expiratory volume over 1 second; BMI = body mass index; CFTR = cystic fibrosis transmembrane conductance regulator; 25OHD = 25‐hydroxyvitamin D.

The participants who did not undergo a DXA scan had similar characteristics to the study group. The mean age of participants mid‐study (2014) was 28.8 (SD = 10.7) years. Of the participants, 53% were male, 91% were white, 43.6% were homozygous, and 82.7% were pancreatic insufficient. The mean FEV_1_% predicted was 75.1 ± 22.3%, mean BMI was 24.4 ± 3.5 kg/m^2^, and 49.1% were on a CFTR modulator.

The type of DXA scanner model varied between patients and included Hologic (Marlborough, MA, USA) Discovery, Hologic Delphi, Hologic QDR4500, Hologic Horizon, and GE Lunar (Madison, WI, USA) Prodigy. All scans reviewed were from central DXA devices.

### Bone mineral density results

A total of 347 DXA scans in 234 individuals were performed within the study period. Of all DXA scans performed, 136 (37.5%) demonstrated normal BMD, 165 (47.6%) demonstrated AR‐CFLBMD, and 46 (13.3%) demonstrated CFLBMD.

**Fig. 1:**
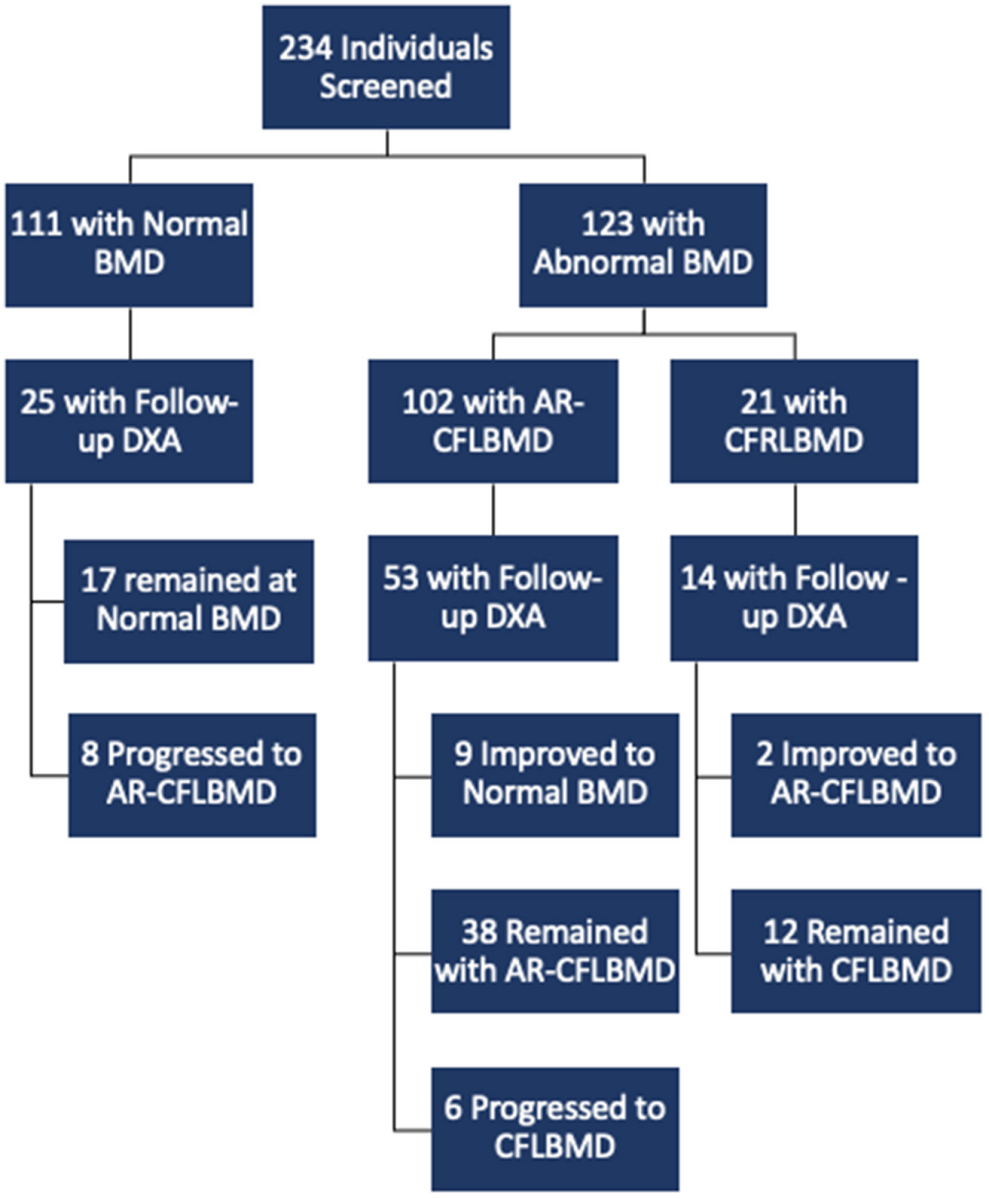
Study flow chart. BMD = bone mineral density; DXA = dual‐energy X‐ray absorptiometry; AR‐CFLBMD = at risk for progression to cystic fibrosis–related low bone mineral density; CFLBMD = cystic fibrosis–related low bone mineral density.

At time of initial DXA scan, 123 (prevalence 52.6%; 95% CI 46.0–59.1) individuals had lower than expected BMD. Of these individuals, 102 (prevalence 43.6%; 95% CI 37.1–50.2) had AR‐CFLBMD and 21 (prevalence 9.0%; 95% CI 5.6–13.4) had CFLBMD (Fig. 1). Of the 111 individuals with normal BMD at initial DXA scan, 25 (22.5%) had at least one follow‐up DXA scan for the purpose of comparison analysis over time: 8 (32.0%) progressed to CFLBMD and 17 (68.0%) remained at a normal BMD. Of the 102 individuals with AR‐CFLBMD at initial DXA, 53 had a follow‐up: 6 (11.3%) progressed to CFLBMD, 9 (17.0%) returned to normal BMD, with the remainder maintaining AR‐CFLBMD. Of the 21 with CFLBMD at initial DXA scan, 14 (66.7%) had a follow‐up DXA: 2 (14.3%) returned to AR‐CFLBMD (Fig. 1). For those with follow‐up DXA scans, the average time interval between scans was 2.79 years. A total of 42.1% of individuals with repeat scans had scans using the same make and model, 15.7% had scans that used different models, and 42.1% had repeat scans in which the make and model were not recorded.

### Risk factors assessment

Results of GEE‐based Poisson regression evaluating the risk of low BMD are presented in Table 2. After adjustment for other covariates, older age (RR = 1.01; 95% CI 1.00–1.01) and male sex (RR = 1.32; 95% CI 1.04–1.66) were associated with increased risk of lower than expected BMD, whereas higher FEV_1_% predicted (RR = 0.99; 95% CI 0.99–1.00) and BMI (RR = 0.97; 95% CI 0.94–1.00) were associated with lower risk for lower than expected BMD.

**Table 2:** Risk Factor Identification for lower than expected BMD

	Unadjusted RR (95% CI)	P value	Adjusted RR (95% CI)	P value
Age (years)	**1.01** **(1.003, 1.018)**	**0.004**	**1.01** **(1.004, 1.023)**	**0.005**
Male sex	**1.33** **(1.047, 1.682)**	**0.019**	**1.32** **(1.043, 1.664)**	**0.021**
Race, non‐white	1.29 (0.897, 1.851)	0.171	1.42 (0.960, 2.112)	0.079
Genotype				
Homozygous F508del	Ref		Ref	
Heterozygous F508del	1.02 (0.791, 1.309)	0.892	1.03 (0.788, 1.358)	0.809
Other	1.08 (0.742, 1.569)	0.689	1.21 (0.801, 1.817)	0.370
Pancreatic insufficiency	1.16 (0.818, 1.647)	0.403	1.06 (0.741, 1.525)	0.739
FEV_1_ % predicted	**0.99** **(0.984, 0.993)**	**<0.001**	**0.99** **(0.987, 0.996)**	**<0.001**
BMI (kg/m²)	**0.97** **(0.947, 0.9999)**	**0.049**	**0.97** **(0.939, 0.995)**	**0.022**
CFTR modulator use	1.16 (0.911, 1.470)	0.233	1.25 (0.971, 1.599)	0.084

RR = relative risks, CI = confidence interval, Ref = reference, FEV_1_ = forced expiratory volume over 1 second, BMI = body mass index, CFTR = cystic fibrosis transmembrane conductance regulator

## Discussion

Despite the growing appreciation for increased risk of low BMD in adults with CF, bone health screening often receives less attention from CF clinicians than other CF issues. In fact, large patient registries report the prevalence of lower than expected BMD to be only half of what has been estimated by smaller cohort studies.^(^ ^1^, ^2^, ^3^, ^4^ ^)^ This discrepancy suggests that clinicians are not sufficiently screening their patients (or patients are not completing the DXA scans) despite current CFF and ECFS guidelines recommending universal screening for all CF adults.^(^ ^8^, ^9^ ^)^ The results of this study showed that the prevalence of CF adults with lower than expected BMD in our cohort (52%) was more than twice that reported in national registries. We also found that while older age, male sex, lower BMI, and lower FEV_1_% predicted were associated with lower than expected BMD, some people without these characteristics also developed lower than expected BMD. Some of the discrepancies in reported prevalence may be due to the complexity of CF care, which causes attention to be focused on more prominent health challenges, like repeated pulmonary exacerbations and need for intravenous antibiotics and hospitalizations, malnutrition, and inability to maintain a healthy BMI, CFRD, and CFLD. At times, DXA scans may not be able to be obtained at the facility where the person with CF goes for their CF care because of insurance reasons, and adherence to getting the test at another facility can be low. However, low BMD is important to treat in CF adults because it is associated with higher morbidity^(^ ^11^, ^12^ ^)^ and can lead to significant challenges if patients need future lung transplantation.^(^ ^13^ ^)^ CFLBMD can be a relative contraindication to lung transplantation given post‐transplant treatment includes long‐term glucocorticoids, which can worsen BMD. With improved survival, people with CF are living longer, and appropriate attention should be paid to diagnosis and management of lower than expected BMD.

While the WHO and ISCD classify osteoporosis and osteopenia as described above, in this study we chose particular nomenclature and *T*/*Z*‐score values to align with the intent of CFF bone care guidelines: to promote adequate follow‐up for those at risk of disease progression and to provide more intensive treatment for individuals with more severe bone disease.^(^ ^8^ ^)^ We have retained the ECFS term “CFLBMD” to describe DXA *T*/*Z* scores ≤2.0, and have decided on the term “AR‐CFLBMD” to describe DXA scores *T*/*Z*‐scores between −1.0 and −2.0. This is to highlight that CFF care guidelines recommend that those within this *T*/*Z*‐score range should be monitored more closely and potentially treated to protect against disease progression.^(^ ^8^ ^)^

Having greater confidence in the true prevalence of lower than expected BMD and the strength of its associated risk factors would help identify a CF population in which DXA scans could be considered essential. Our study aimed to contribute in both of these areas, utilizing clinical experience from 2010 to 2018 at one of the largest Adult CF Programs in the United States. The results of this study demonstrated that the prevalence of lower than expected BMD in the adult CF population is much higher than reported in national CF patient registries. Based on our DXA scan analysis, the estimation of lower than expected BMD prevalence is 52.4%, which is much higher than the 25% prevalence reported in the registries. Although significant associations were found between some analyzed risk factors and lower than expected BMD (particularly older age, male sex, and those with lower BMI and FEV_1_), individuals with lower than expected BMD have a broad range of clinical characteristics. Therefore, it may not be possible to predict who will develop lower than expected BMD. These conclusions support the importance of universal bone health screening of all CF adults.

Four risk factors were found to have statistically significant associations with lower than expected BMD. The presence of these factors in an individual should suggest a greater attention to screening. Risk factors associated with lower than expected BMD included older age, male sex, lower BMI, and lower FEV_1_% predicted. Although low BMI, lower lung function, and older age are all recognized as likely risk factors, male sex may appear surprising as studies in the general population have shown that females have a higher risk of developing low BMD.^(^ ^23^ ^)^ It is important to note that other recent studies have also identified male sex as a risk factor for lower than expected BMD in the CF population.^(^ ^24^ ^)^ It is unclear if males with CF who may have a higher risk of developing lower than expected BMD are more likely to be screened or if all males with CF are at higher risk. This highlights the danger of projecting data from the non‐CF population onto CF adults. For example, assuming that only older females have a high risk of lower than expected BMD and therefore should be screened may cause fewer CF males to be screened.

This study provides insight into the progressive nature of CF‐related bone disease. Of people who were initially found to have normal BMD, 32% subsequently progressed to AR‐CFLBMD over an average of 8 years. Of those with initial AR‐CFLBMD, 11% progressed to CFLBMD. Current CFF guidelines recommend that people with normal DXA *T*/*Z*‐scores repeat the DXA scan every 5 years, and those with scores between −1.0 and −2.0 have a repeat DXA every 2 to 4 years and those scores ≤ −2.0 have an annual DXA scan.^(^ ^8^ ^)^ Our data showing continued progression highlights the importance of continued monitoring, as recommended in the guidelines, in addition to early intervention and treatment when needed.

Vitamin D deficiency is common in people with CF^(^ ^1^, ^3^, ^4^ ^)^ and can contribute to bone loss, and repletion of 25OHD is recommended.^(^ ^8^ ^)^ Although we did not look at individual levels of 25OHD in patients that progressed, our clinical care program aggressively treats 25OHD deficiency as attested by the normal mean serum level of the study population. We did not include 25OHD levels in our analysis because of the wide seasonal variation in serum levels.

We found that 9.8% of individuals were on bisphosphonate therapy before their first DXA scan and that 7.6% of individuals started bisphosphonate in between their first and subsequent scans. This may have affected their baseline and follow‐up BMD status. It is our practice to refer all patients at our center with a diagnosis of CFLBMD to a dedicated endocrinologist who treats our patients who have CFRD as well as low BMD.

It is important to note that the study population had a very limited exposure to highly effective CFTR modulation, as very few people with CF were on highly effective CFTR modulators during the study period. CFTR modulators are drugs that work to help correct the dysfunctional CFTR protein, improving its functional abilities. They are effective at improving disease in areas in which the CFTR protein is present: in the lungs, pancreas, GI tract, skin, as well as cells involved in osteoblast formation.^(^ ^25^ ^)^ There are four CFTR modulators approved on people with CF, but eligibility depends on the type of mutations an individual with CF has. The first CFTR modulator, ivacaftor, was approved in 2012. Subsequently, lumacaftor/ivacaftor and tezacaftor/ivacaftor were approved. Of note, a fourth CFTR modulator was approved after the study period: elexacaftor/tezacaftor/ivacaftor in 2019.^(^ ^8^ ^)^ Both ivacaftor and elexacaftor/tezacaftor/ivacaftor are considered highly effective modulator therapy (HEMT), whereas lumacaftor/ivacaftor and tezacaftor/ivacaftor are not.^(^ ^26^ ^)^ Treatment with each of these modulators is recorded separately in this study because of the difference in clinical efficacy. HEMT is shown to provide a greater increase in health outcomes including FEV_1_% predicted and BMI compared with the other modulators. Modulator therapy may affect bone health of those with CF directly by increasing CFTR function in osteoblast formation and indirectly through improving nutritional status.^(^ ^27^ ^)^ The majority of the future adult CF population will benefit from highly effective CFTR modulation, as now 90% of people with CF in the United States are eligible for HEMT. Because elexacaftor/tezacaftor/ivacaftor was not available during the time frame of our study, our study group may not be fully representative of the future CF experience. These CFTR modulators may help to stabilize or even improve bone health, though this is not known. Until more data regarding this are available, however, continued attention to bone health is necessary. This attention may also help provide insight into the effects of successful CFTR modulation on CF bone health.

Although we only analyzed our adult population for this study, we would like to highlight important considerations in the assessment of children and adolescents. Current CFF guidelines recommend DXA screening in children >8 years old if they have any of the following risk factors: <90% ideal body weight, FEV_1_ < 50% predicted, glucocorticoids of ≥5 mg/d for ≥90 d/yr, delayed puberty, or a history of fracture. European and French guidelines recommend DXA scans in all children 8 to 10 or 8 years of age, respectively. Additionally, bone density is recommended to be measured at the spine and/or total body less head in children and adolescents. Importantly, as bone mass should only be increasing with time in children and adolescents, any finding of bone loss over time is concerning in this patient population and merits consideration for pharmacologic treatment. Although some studies have found that children and adolescents with CF have a higher prevalence of low BMD, other research has suggested that areal BMD development remains similar in healthy children and those with CF,^29^ as long as they receive adequate medical care. Further research clarifying these results may be useful in preventing low BMD among CF adults.

*T*/*Z*‐scores of all study participants were taken at the lumbar spine, total hip, or femoral neck. CFF guidelines state that either *T*‐score or *Z*‐score can be used between the ages of 18 and 30 as the scores are very similar in this age range; over the age of 30 years, *T*‐scores are preferred. Our data collection followed this recommendation. To improve our analysis and clarify those most at risk for developing fractures, further assessment of our cohort using a fracture risk assessment tool may predict which individuals will benefit most from therapeutic intervention.

There are several limitations to the present study. First, 126 (35%) of patients seen at our center did not have a DXA scan during the study period and selection bias may have been introduced. Individuals who did not undergo a DXA scan tended to be younger, male, of normal weight, and have less severe lung dysfunction. However, even considering a best‐case scenario in which all individuals who did not have a DXA scan did have normal BMD, the prevalence of lower than expected BMD would be 34.2% and still above the national estimate. For those with repeat scans, some DXA exams were taken at different locations for patient flexibility. Some of the scans did not document the make and model of scanner used for the exam. Current ISCD best practices recommend that all scans be taken by the same machine and scanner operator for more precise comparison.^(^ ^28^ ^)^ This limitation may affect the diagnosis of AR‐CFLBMD or CFLBMD considering potential discrepancies in BMD and/or *T*/*Z*‐score results that may occur between different DXA machines and scanner operators.

It is known that postmenopausal women are at higher risk of lower than expected BMD than the normal population,^(^ ^22^ ^)^ making it difficult to distinguish between age‐ and menopause‐related low BMD versus CF‐related low BMD. Although we were unable to discriminate low BMD because of menopause from low BMD due to CF, there are only 6 female study participants over the age of 55 years, indicating that this is a very small percentage of our population.

Additionally, the usage of medications like calcium supplementation was not collected because of the complexity of CF care. Nutritional and lifestyle information, such as food, calcium intake, and exercise were not collected because of the lack of availability of verifiable data. At our center, it is our practice that all patients, especially those with lower than expected BMD, are provided nutritional guidance and calcium supplements when necessary. Each patient meets with our nutritionist yearly or more often as needed. In terms of quantifying bone turnover, markers of bone turnover are not routinely obtained at our center and therefore we would not be able to correlate changes in bone turnover markers to changes in bone mineral density. There also may be other, non‐CF‐specific risk factors for fragility factors that we did not consider in this study.

A final limitation, which is common to all single‐center studies, concerns the generalizability of the results from our center to other CF care centers. The Johns Hopkins Adult CF center is a recognized center of excellence, which implies that the care provided to patients in this cohort may be more robust than that elsewhere. The generalizability of our reported prevalence may suffer due to this factor; however, this suggests that other centers may have even higher prevalence of poor bone health—further demonstrating our hypothesis that low BMD is underreported.

The prevalence of CF adults with lower than expected BMD in this study was found to be more than twice that reported in national registries, suggesting that the actual prevalence in CF adults may be much higher than currently reported. Although low BMI, low lung function, older age, and male sex were found to be associated with developing lower than expected BMD, individuals without these risk factors were also found to have lower than expected BMD. This supports the importance of universal bone health screening of all CF adults.

## Disclosures

All authors state that they have no conflicts of interest.

## Author Contributions

**Rebecca L Boyle:** Conceptualization; data curation; writing ‐ original draft; writing ‐ review and editing. **Kevin J Psoter:** Conceptualization; formal analysis; methodology; writing – original draft; writing – review and editing. **Christian A Merlo:** Conceptualization; writing – review and editing. **Aniket R Sidhaye:** Conceptualization; writing – review and editing. **Noah Lechtzin:** Conceptualization; writing – review and editing. **Shivani Patel:** Data curation; formal analysis; writing – review and editing. **Kristina Montemayor:** Writing – review and editing. **Alexandra Horne:** Writing – review and editing. **Natalie E West:** Conceptualization; investigation; methodology; project administration; writing – original draft; writing – review and editing.

### Peer Review

The peer review history for this article is available at https://publons.com/publon/10.1002/jbm4.10666.

## Data Availability

The data that support the findings of this study are available on reasonable request from the corresponding author.
